# Assessing a Syndemic of Discrimination, Material Insecurity, Depression, Substance Use, and Violence Among Sexual and Gender Minorities in Nigeria Using Mixed Methods

**DOI:** 10.1007/s10461-025-04861-1

**Published:** 2025-08-23

**Authors:** Rodman Turpin, Megan E. Mansfield, Typhanye Dyer, Andrew Mitchell, Chama John, Ruxton Adebiyi, Uchenna Ononaku, Christiana Katu, Jumoke Aigoro, Abayomi Aka-Bashorun, Sylvia Adebajo, Manhattan Charurat, Rachel Sullivan Robinson

**Affiliations:** 1 Department of Global and Community Health, College of Public Health, George Mason University, Fairfax, VA USA; 2 Institute of Human Virology, University of Maryland, Baltimore, MD USA; 3 Department of Epidemiology and Biostatistics, School of Public Health, University of Maryland, College Park, MD USA; 4 Institute of Human Virology, Abuja, Nigeria; 5 International Centre for Advocacy and Right to Health (ICARH), Abuja, Nigeria; 6 School of International Service, American University, Washington, D.C. USA

**Keywords:** HIV, Syndemic, Mixed methods, LGBT, Black

## Abstract

Sexual and gender minority people (SGM) in Nigeria experience disproportionate HIV burden, with an HIV prevalence four to ten times higher than the national average. Better understanding the factors that create HIV vulnerability in this population is important for designing effective interventions, particularly in a context largely hostile to SGM. We assessed a conceptual model describing a syndemic of discrimination, material insecurity, depression, substance use, intimate partner violence, and police and other violence among SGM in Abuja, Nigeria. As part of a larger, longitudinal study examining noncommunicable disease outcomes within this population, we conducted a mixed methods analysis using both quantitative intake data (*n*=515) as well as data from three focus groups (*n*=36), collected from July 2023 through May 2024. We tested for intercorrelations among syndemic components, and associations between a cumulative syndemic index and HIV status using modified Poisson regression. We also conducted a convergent qualitative assessment of the conceptual model in three focus group discussions. Finally, we examined co-prevalence of syndemic components highlighted in our qualitative findings. There were consistent intercorrelations among syndemic components, supporting the presence of a syndemic. After adjustment for sociodemographic factors, every quartile-unit increase in the syndemic index was associated with an 18% increase in prevalence of HIV (aPR=1.18, 95% CI 1.07, 1.29). Additionally, our qualitative findings highlighted relationships between discrimination, material insecurity, and depression as especially relevant among this population. When using our quantitative data to examine the co-prevalence of pairs of syndemic components identified as particularly salient in our qualitative analyses, nearly every relationship was significantly stronger than expected. We found strong evidence of a syndemic of discrimination, material insecurity, depression, substance use, intimate partner violence, and police and other violence among SGM in Abuja, Nigeria as salient to the health outcomes of SGM in Nigeria. Overall, our findings highlight the presence of a multilevel syndemic that informs multilevel intervention targets. Interventions must target not simply the individual level, but also incorporate larger scale social and structural change efforts.

## Introduction

HIV in Nigeria remains a critical area for public health efforts. Based on both national surveillance and predictive modeling, the estimated national HIV prevalence in Nigeria among adults aged 15–49 years ranges from 1.3 to 2.1%, equivalent to about 1.5 to 2 million people living with HIV [1, 2]. Notably, sexual and gender minority people (SGM) in Nigeria are disproportionately vulnerable to HIV; this includes men who have sex with men (MSM), gay, bisexual, and other people with sexually minoritized identities, and transgender women [3, 4]. HIV prevalence among Nigerian MSM is estimated to be 23%, ten times higher than the national average [1]. Among Nigerian transgender women (TGW) and other SGM sampled through community-based health centers offering SGM-friendly services, HIV prevalence ranged from 44 to 66% [5]. Factors that have been associated with HIV among SGM populations include depression, discrimination, violence, and substance use [6–8], which often co-occur with one another, substantially affecting health outcomes for this population. These disproportionately high prevalence rates, as well as the high prevalence of co-occurring conditions, highlight the need for better understanding the co-occurrence of factors that impact HIV vulnerability in this population.

**Fig. 1:**
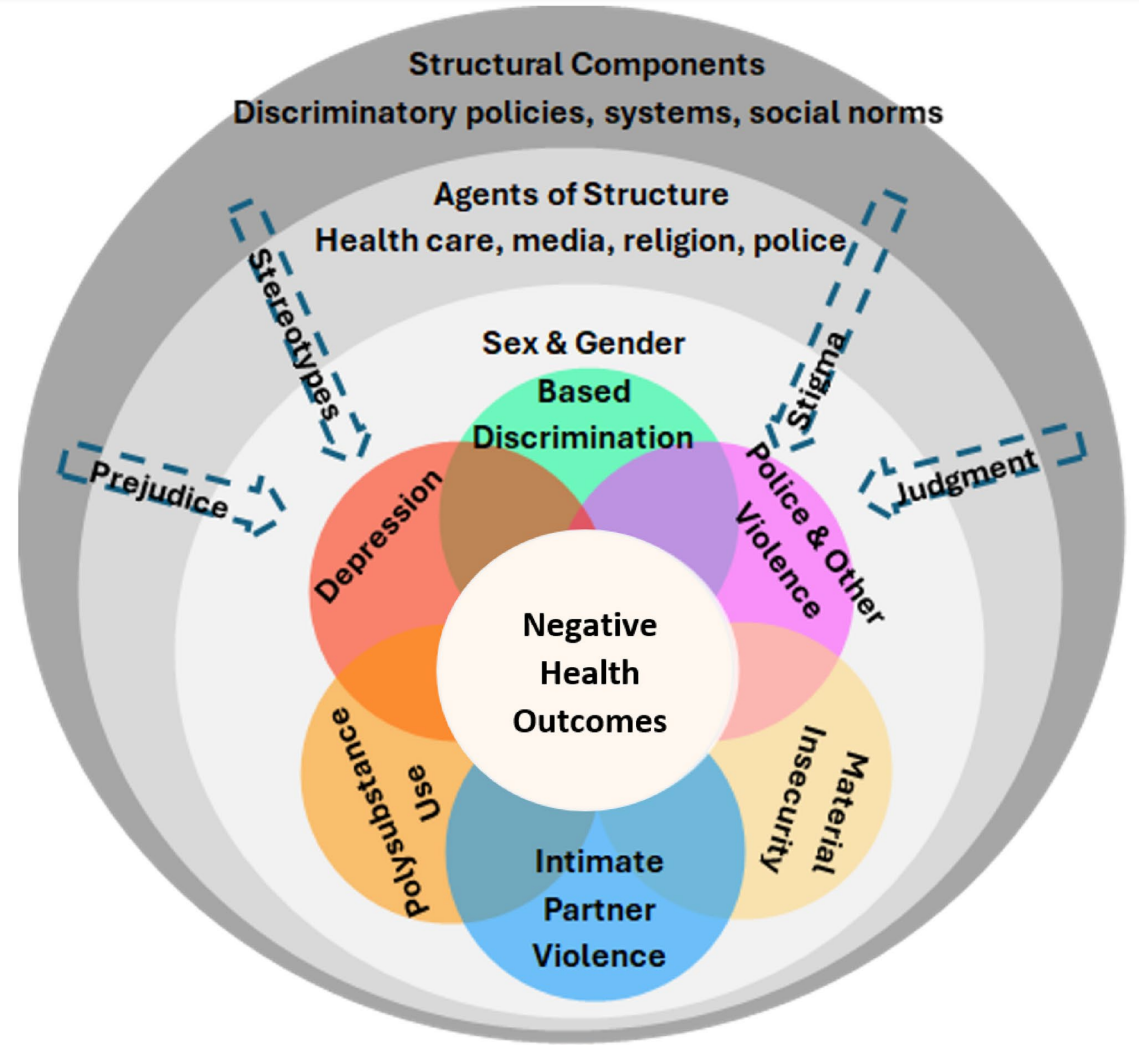
Syndemic framework conceptual model

Syndemic theory posits that risk factors cluster because of common social and structural drivers, which amplify the negative impact of these risk factors on health outcomes, such as HIV prevalence, diabetes, and others [9]. This theory has been utilized to more effectively elucidate synergistic HIV risk factors among marginalized communities of SGM, including Black SGM in the United States [6–8]. Our conceptual framework (Fig. 1) illustrates how six different individual-level factors particularly relevant to the lived experiences of SGM (sex and gender-based discrimination, depression, intimate partner violence, police and other violence, material insecurity, and polysubstance use) interact to form a syndemic associated with adverse health outcomes in this population, particularly related to HIV. Importantly, structural factors impact and affect the clustering and interactions of individual-level factors. Structural factors identified in our conceptual framework include discriminatory polices (e.g., the Same-Sex Marriage (Prohibition) Act, the Nigerian Criminal Code) and social norms regarding marriage, childbearing, and gender. These structural factors facilitate discrimination by health care providers, the media, religious leaders, law enforcement, family members, and the overall community and there is substantial literature supporting the relationships between these factors in related populations [10–13]. For example, several studies among Black MSM have demonstrated relationships between discrimination and depression [10–14], intimate partner violence and substance use [15], stigma and mental health [13, 16], and socioeconomic insecurity and depression [17, 18]. The specific legal, social, and economic context of Nigeria related to the criminalization of same-sex sexuality, strong social norms around childbearing, and the high poverty rates, makes this syndemic uniquely relevant to the health outcomes of the Nigerian SGM population [11].

The purpose of this study is to assess the presence of a syndemic of discrimination, material insecurity, depression, substance use, intimate partner violence, and police and other forms of violence among SGM in Nigeria using a convergent mixed methods approach. We tested for quantitative evidence of interrelationships of the components identified in our framework and associations with HIV status. We also qualitatively explored relationships between these components using focus group discussions (FGD). Finally, we assessed with the quantitative data intercorrelations of pairwise components highlighted by our qualitative findings. We hypothesized that this syndemic characterized by our conceptual model (see Fig. 1) would be associated with positive HIV status among SGM in Nigeria, and that qualitative findings would provide insight into how the different elements of this syndemic interrelate to impact the health of the SGM community.

## Methods

### Study Setting

This convergent mixed method study is part of a prospective longitudinal cohort study (5R01HL165686-02) examining noncommunicable disease (NCD) outcomes among SGM in Abuja, Nigeria. The overall aims of the parent study are to (1) examine associations between a multilevel syndemic and both HIV and NCD outcomes, (2) assess the influence of structural-level components on HIV and NCDs, and (3) determine the extent to which syndemic components increase risk of disengaging from care for HIV and NCDs. This study was conducted at the TRUST Clinic in Abuja, Nigeria, established in 2012 through a tripartite partnership among the nonprofit community-based organization, International Center for Advocacy and Rights to Health (ICARH), the Institute of Human Virology Nigeria, and the University of Maryland Baltimore [19]. As a result of this well-established collaboration, ICARH has recruited a cohort of nearly 2,800 SGM and rigorously evaluated the benefits of HIV test and treat within the secure environment of the community center. The TRUST Clinic provides broad HIV care and prevention as well as other health services to the SGM community and is embedded within the community-based organization ICARH. ICARH conducts advocacy programs, research, and human rights activism. The clinic has an on-site laboratory for HIV viral load monitoring and an in-house pharmacy which dispenses antiretroviral therapy, pre-exposure prophylaxis, and post-exposure prophylaxis as well as other routine medicines. These HIV prevention and management services are provided to clients by a team of SGM-friendly nurses, a pharmacist, a social worker, the HIV testing service team, and community extension workers. We collected all data from July 2023 through May 2024.

### Quantitative Measures

Quantitative data come from intake surveys. Syndemic exposures included depression using the PHQ-9 (continuous) [20], any cigarette use (yes, no), any stimulant use (yes, no), a 17-item index of material insecurity (continuous) [21], any intimate partner violence (yes, no), any other interpersonal violence (yes, no), a three-item index of experienced sexual/gender discrimination from family (yes, no), a five-item index experienced sexual/gender discrimination in healthcare settings (yes, no), a three-item index experienced sexual/gender discrimination or harassment from police (yes, no), and a nine-item index HIV stigma (continuous). All discrimination and stigma items were adapted from the American Men’s Internet Survey of Men Who Have Sex With Men in the United States [22]. Our 17-item measure of material insecurity is a composite of a nine-item measure of financial insecurity and an eight-item measure of nutritional insecurity [21]. We combined these two sets of measures due to both strong conceptual similarities (e.g., some of the financial insecurity items also reflect nutritional insecurity) but also very strong covariance between the two indices. Additionally, we kept the three constructs for SGM discrimination distinct, as these had varied relationships with other syndemic components. All components were scaled to range between 0 and 1 (i.e., scaled in percentage) to allow for equivalent weighting of items. For all scales, we subtracted the original scale minimum, then divided by the original scale maximum minus the original scale minimum. This transforms all scales to range between 0 and 1. For example, the PHQ-9 ranges from 0 to 27, so we subtracted 0, then divided by 27 minus 0, which simplified to dividing all values by 27. This resulted in a transformed scale that ranged from 0 to 1. Percentage scaling allows for items to be compared visually with more ease, with means for continuous items and proportions for binary items having the same range. Our primary outcome for assessing the syndemic was HIV status (negative, positive) confirmed through biological testing. Covariates included age (18–24, 25–29, 30–34, 35+), highest education level (less than secondary, senior secondary school, more than senior secondary), personal income (Naira:) in the last month (less than 20,000, 20,000 to 59,999, 60,000 or more), and sexual identity (bisexual, gay, other). We selected covariates based on associations with HIV status and vulnerability identified in the literature.

### Bivariate Analyses

We tested for associations between our syndemic index and ordinal covariates (age, education, income) using Spearman rank-sum correlation. Similarly, we tested for associations between our syndemic index and our binary outcome (HIV status) using a point-biserial Spearman rank-sum correlation. We also tested for associations between sexual identity and our syndemic index using a Kruskal-Wallis test. Finally, we tested for intercorrelations between all of our syndemic components using both Spearman rank-sum correlation (between two continuous/ordinal components), point-biserial Spearman rank-sum correlation (between binary and continuous/ordinal components), and Phi correlations (between two binary components). We also present syndemic components associated with HIV status individually.

### Regression Analyses

We used modified Poisson regression with robust standard errors to test associations between our syndemic index and HIV status. This method is useful for generating prevalence ratios for binary outcomes and allows for inclusion of more confounders than log-binomial modeling. We generated unadjusted models and models adjusted for age, education, and personal income. We did not include sexual identity in models due to stability difficulties related to the unusually small “other” category. For all models we generated ratio estimates and 95% confidence intervals for associations between quartile-unit increases in the index and HIV status. The total range of the index was 0 to 10, and a quartile unit increase was 3 units, as this was half the interquartile range rounded to the nearest whole number. So each estimate in our regression models reflects a 3-unit syndemic index increase. We use quartiles as this reflects a more meaningful change in the index rather than a 1-unit increase, which would be overly small given the numerous constructs included. The quartile increase also roughly approximates a standard deviation, a commonly used unit measure for index differences, but without relying on assumptions of normality likely to be violated. Fit and variance metrics for models are also included. Additionally, we utilized a syndemic index with the HIV stigma component removed, to determine if there was a substantial difference in the association with HIV status without this component.

### Quality Assurance

Missingness for all items was low (less than 10%), with most items having less than 5% missingness. Given the low level of missingness, we employed maximum likelihood imputation to impute missing data. Post-imputation, we retained all observations (*n* = 515). Analysis of Cook’s distances and leverages revealed no significant outliers. Additionally, variance inflation factors of less than 5 for all models indicated no evidence of intercollinearity. All quantitative analyses were conducted in SAS 9.4 [23].

### Qualitative Analyses

In addition to the analysis of intake survey data, we also conducted a convergent qualitative assessment of the conceptual framework, and its six components. Participants from the Syndemics Study who were enrolled as of September 2023 (*n* = 70) were randomly selected for participation in the first two FGD (*n* = 24). A third focus group (*n* = 12) was conducted with purposeful sampling of TGW resulting in three FGD with 36 participants across them. Each of the three FGD included 8–13 participants. Participants provided informed consent individually prior to participating in the FGD and received transportation funds as well as lunch after the FGD was completed. During the FGD, we prompted participants to discuss their conceptualizations of health; structural, community, and individual influences on health; and experiences and observations related to NCDs. Each FGD lasted about two hours. The FGD were audio recorded, transcribed verbatim and – as needed – simultaneously translated into English (from either Hausa or Pidgin). Two members of the research team independently coded the transcripts (MM and RSR) using a hybrid deductive-inductive coding method and the qualitative analysis software NVivo. We began with the creation of an initial codebook based predominately on deductively identified codes from the FGD guide and syndemic framework. We coded all three FGD using the initial codebook and then discussed points of disagreement and agreement in coding. This resulted in a refined codebook that also took into consideration patterns that emerged inductively from the data. We then independently coded the three transcripts again and discussed disagreements until agreement was reached. After coding was completed, themes were identified and organized using axial coding.

### Co-occurrence and Sensitivity Analyses

Following both initial quantitative and qualitative analyses, we assessed with the quantitative data the co-occurrence of syndemic components highlighted in the qualitative findings. Additionally, we conducted sensitivity analyses collapsing substance use into one factor, and discrimination into a single factor, as these were the only two constructs with more than one component in the index. We compared results for this modified index to the unmodified index.

### Ethics

This study received ethics approval from the institutional review boards of the University of Maryland, Baltimore, USA and the National and Federal Capital Territory Health Research Ethics Committees, Abuja, Nigeria. Separate written consent was provided for the quantitative and qualitative components.

## Results

### Sample and Bivariate Results

**Fig. 2:**
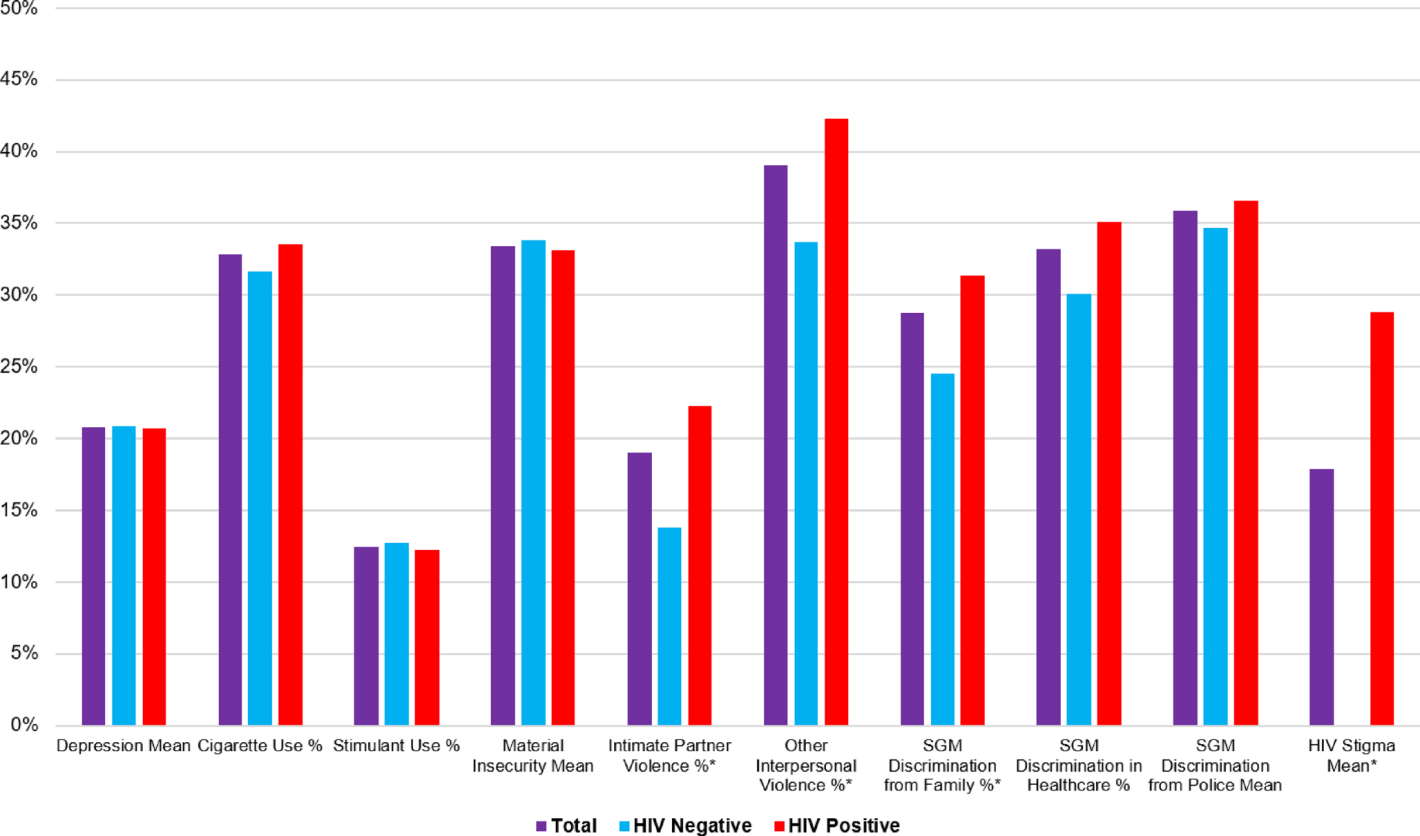
Means and proportions (%) of syndemic components overall and across HIV status among sexual and gender minority people in Abuja, Nigeria (n=515). Groups of factors include Depression (1 factor), Intimate Partner Violence (1 factor), Material Insecurity (1 factor), Other Violence (1 factor), Substance Use (2 factors), Discrimination (3 factors), and Stigma (1 factor). Means are scaled in percentage from 0 to 1 (i.e., 0% to 100%) to be comparable to binary item percentages. *Significant (p<.05) difference between HIV negative and HIV positive participants

Our final sample consisted of 515 participants (Table 1). The median age was 25–29, and the median highest education level was senior secondary school. Two-thirds of the sample reported a personal income of less than 60,000 in the last month. Just over half of the sample identified as bisexual (57.3%), and just over a third identified as gay (37.5%). Nearly two-thirds of the sample was HIV-positive (61.9%). A greater syndemic index was associated with younger age, “other” sexual identity, and positive HIV status. Additionally, consistent intercorrelations among syndemic components existed, supporting the use of a syndemic index (Table 2). Intimate partner violence, other personal violence, and HIV stigma were all associated with positive HIV status (Fig. 2).

**Table 1:** Sociodemographics, HIV status, and syndemic index mean and standard deviation (SD) among sexual and gender minority people in Abuja, Nigeria (*n* = 515)

	Total		Syndemic index	
	*n*	%	Mean	SD
*Total*			2.61	1.83
*Age*				
18–24	135	26.2	**2.67**	**1.86**
25–29	161	31.3	**2.86**	**1.77**
30–34	121	23.5	**2.53**	**1.88**
35+	98	19.0	**2.22**	**1.85**
*Highest Education Level*				
Less than Secondary	44	8.5	2.46	1.64
Senior Secondary School	224	43.5	2.56	1.84
More than Senior Secondary School	247	48.0	2.69	1.89
*Monthly Personal Income*				
Less than 20,000 Naira	127	24.7	2.82	1.97
20,000 to 59,999 Naira	219	42.5	2.61	1.77
60,000 Naira or more	169	32.8	2.45	1.83
*Sexual Identity*				
Bisexual	295	57.3	**2.40**	**1.75**
Gay	193	37.5	**2.83**	**1.83**
Other	27	5.2	**3.28**	**2.57**
*Current HIV Status*				
Negative	196	38.1	**2.36**	**1.63**
Positive	319	61.9	**2.77**	**1.95**

^1^Association with syndemic index tested using Spearman rank-sum correlation. Significant (*p* <.05) values bolded

^2^Association with syndemic index tested using point-biserial Spearman rank-sum correlation. Significant (*p* <.05) values bolded

**Table 2:** Spearman rank-sum correlation matrix of syndemic component factors among sexual and gender minority people in abuja, Nigeria (*n* = 515)

	Cig	Stim	MatI	IPV	Viol	SDF	SDH	SDP	HIVS
Dep	0.07	**0.09**	**0.43**	**0.27**	**0.32**	**0.19**	**0.23**	**0.13**	**0.24**
Cig	–	**0.34**	**0.14**	**0.15**	**0.13**	**0.12**	−0.02	**0.11**	0.07
Stim	–	–	**0.17**	**0.10**	**0.10**	**0.16**	0.03	**0.11**	**0.12**
MatI	–	–	–	**0.21**	**0.26**	**0.12**	**0.15**	**0.09**	**0.22**
IPV	–	–	–	–	**0.43**	**0.39**	**0.24**	**0.24**	**0.22**
Viol	–	–	–	–	–	**0.35**	**0.19**	**0.21**	**0.23**
SDF	–	–	–	–	–	–	**0.15**	**0.20**	**0.30**
SDH	–	–	–	–	–	–	–	0.05	**0.20**
SDP	–	–	–	–	–	–	–	–	0.07

*Dep* = Depression mean, *Cig* = Any cigarette use, *Stim* = Any stimulant use, *MatI* = Material insecurity mean, *IPV* = Any intimate partner violence, *Viol* = Any other interpersonal violence, *SDF* = Any SGM discrimination from family, *SDH* = SGM discrimination in healthcare, *SDP* = SGM discrimination from police, *HIVS* = HIV stigma (HIV stigma correlations measured only in HIV positive subsample *n* = 319)

Intercorrelations tested using Phi correlations (between pairs of binary variables), Spearman rank-sum correlations (between pairs of numeric variables) and point-biserial Spearman rank-sum correlations (between pairs with one binary and one numeric variable). Significant (*p* <.05) values bolded

### Regression Results

In both unadjusted and adjusted regression models, our syndemic index was significantly associated with positive HIV status (Table 3). After adjustment, every quartile-unit increase in the syndemic index was associated with an 18% increase in prevalence of HIV (aPR = 1.18, 95% CI 1.07, 1.29). Notably, estimates were largely similar between adjusted and unadjusted models. Older age was also associated with HIV prevalence. Our socioeconomic covariates were not, though it should be noted that our syndemic index also captures socioeconomic deprivation. Finally, removing the HIV stigma component from the index only slightly attenuated results, though the syndemic index remained both a statistically significant predictor of HIV status and largely interpreted in the same way (aPR = 1.15, 95% CI 1.05, 1.28).

**Table 3:** Unadjusted and adjusted prevalence ratios for associations between the syndemic index and HIV status among sexual and gender minority people in Abuja, Nigeria (*n* = 515)

	Unadjusted	Adjusted
*Syndemic Index*	**1.14 (1.04, 1.26)**	**1.18 (1.07, 1.29)**
*Age*		
18–24		Reference
25–29		**1.53 (1.20**,** 1.94)**
30–34		**1.84 (1.46**,** 2.33)**
35+		**2.05 (1.62**,** 2.59)**
*Highest Education Level*		
Less than Secondary		0.81 (0.62, 1.07)
Senior Secondary School		0.98 (0.85, 1.12)
More than Senior Secondary School		Reference
*Monthly Personal Income*		
Less than 20,000 Naira		0.92 (0.75, 1.11)
20,000 to 59,999 Naira		1.07 (0.93, 1.23)
60,000 Naira or more		Reference
*Fit Statistics*		
Aikake Information Criterion	717.34	672.28
Corrected Aikake Information Criterion	717.39	672.72
Bayesion Information Criterion	730.07	714.72
Coefficient of Variation	78.08	74.36

Syndemic index estimates presented for a quartile range increase. Adjusted models are adjusted for all factors shown. Significant (*p* <.05) estimates bolded

### Qualitative Results

Participants’ reported ages ranged from 19 to 40 years and the majority (*n* = 22, 61%) identified as men, with the remainder self-identifying as either women (*n* = 12, 33%) or transgender (*n* = 2, 6%). Additionally, of the participants who responded, a plurality identified as gay (*n* = 15, 42%) with the remainder identifying as bisexual (*n* = 9, 25%), queer (*n* = 3, 8%), or something else (*n* = 4, 11%). The FGD participants thus roughly mirrored the broader study population included in the quantitative analysis, although with a lower percentage identifying as bisexual and a higher percentage identifying as women due to our purposive sample of TGW for one FGD.

To parallel the bivariate analysis described above, we conducted a frequency analysis of the co-occurrence of syndemic factors (Table 4). When prompted with the six factors constituting the syndemic, participants discussed depression and material insecurity the most, followed by substance use and discrimination. They mentioned violence (interpersonal or at the hands of the police) somewhat less. This analysis revealed that participants identified connections between all pairs of syndemic factors, with more frequent references to the links between material insecurity and three other factors (depression and substance use in particular, but also discrimination), as well as to connections between depression and two other factors (substance use and discrimination).

**Table 4:** Qualitative coding Co-Occurrence frequency analysis

	1	2	3	4	5	6
1. Depression	64	5	*17*	6	*18*	*12*
2. IPV		16	5	1	4	1
3. Material Insecurity			68	2	*16*	*10*
4. Violence				16	4	4
5. Substance Use					34	5
6. Discrimination						29

Italic values indicate most frequently co-occurring syndemic factors

### Material Insecurity

Many Nigerians face material insecurity, including members of the SGM community. Approximately 40% of Nigerians live below the national poverty line, and two thirds are multidimensionally poor [24]. As in most of the world, economic hardships followed Nigeria’s COVID lockdown, as did spiraling inflation. Nigeria has also uniquely faced currency devaluation, and everyday life has become significantly more expensive in recent years, particularly with the removal of a fuel subsidy following the change in presidential administration in 2023 [25]. SGM are at additional risk of material insecurity given discrimination faced in securing employment and housing [26].

Participants understood material insecurity as preceding depression, often with discrimination at the root of material insecurity. For example, “I have a friend, who lost his job because his boss got to know that he is queer. Now, this person lost his job, lost his source of income, and lost everything. It got to the stage; this guy was depressed” (FGD1, unidentified participant). Or, as someone else described, “When you are discriminated, you might not be able to work. Like you cannot go and apply for jobs in an organization because when you get there, they say, ‘This person is gay. You can’t employ a gay person to work for us.’ So you won’t really have the boldness to go and seek for jobs. When you are discriminated, you get depressed” (FGD2, participant #8). These experiences were particularly acute for SGM who did not conform to gender expectations. For example, “You cannot go to a place and look for a job if you as a feminine person as a guy.. there are so many people, they cannot look for job because their papers are still reading their biological birth” (FGD3, participant #2).

Others noted social norms at the root of material insecurity, with SGM pressured into marriage and having children, not being able to afford the associated responsibilities, and falling into depression. “Nigeria, [these] days of course, nobody’s willing to increase your pay – you work and not get paid, and that’s how it is. But.. then children come in.. and things are not there in place to take care of everything, you start breaking down, mentally” (FGD1, participant #9). Someone else noted, “For the Naira and depression, for somebody like me, it fucked with my mental health. If I’m broke, like it.. gives me this boredom of staying indoors.. I don’t want to see anybody because I don’t have money to go out” (FGD2, participant #10). Or, as another participant put it, “Without having finance, you cannot be able to live the life you want to live, and then will lead into depression” (FGD3, participant #2).

### Discrimination

Participants’ accounts demonstrated multi-faceted experiences with discrimination related to both sexual orientation and gender identity, and associated discriminatory experiences with depression and other mental health issues, including anxiety. Social norms writ large, often enacted by family members as well as religious leaders, allowed for the articulation of discrimination. Participants particularly noted the challenge of meeting expectations around marriage to a woman. “‘Who is your girlfriend?’ is a social norm in our society. Like, when you get to a certain age, and the next question is that, ‘Ah, what’s up? We have not seen you with any girl!’ And before you know it, you will be forced to have a girlfriend.. you’re starting to force yourself to do those things you do not want to do, and breaking down already” (FGD1, participant #6). As someone else described it, “Growing up in a very homophobic environment, where you are judged for basically everything you do, even just for breathing, you will begin to question your life, question everything that you do at that point in time, you will not have anyone to talk to because you’re trying to put up this facade of being straight.. Such situations can lead one into depression” (FGD1, participant #5). Focusing on Nigeria’s laws criminalizing same-sex marriage, one participant noted, “You can’t, like, be with your partner outside. It’s not allowed in this country.. And it has caused fear, I mean scared, like not being free to express my own self” (FGD2, participant #4). Another stated, “With that law, anxiety comes in. Because the fear of going to jail, the fear of being arrested, the fear of being molested by police” (FGD2, participant #8). Participants identified specifically how stigma and discrimination stemming from religion could lead to depression. For example, “When you feel you are not really doing the will of God, you just want to be on yourself, you isolate yourself from friends of the [SGM] community. I think it affects us mentally and it gets us into depression” (FGD2, participant #8). As someone else put it, “Society causes depression because when you’re not treated fairly, you get this injustice, be it from the clinic center, be it from the police station, be it from your church or be it in any peer group. That is when you go back home and you enter into the house of depression” (FGD2, participant #10).

### Substance Use

Participants described high rates of substance use in the SGM community. In particular they identified substance use as a maladaptive coping mechanism for depression, discrimination, and material insecurity. They observed that in the long-term, substance use makes most people’s situation worse. As one participant indicated, “Substance use is very common in the community. Some people think it is a way to cure depression, but it’s killing us faster than the depression will even kill us” (FGD1, participant #9). Another participant explained a link with police violence: “When it comes to the brutality from the policemen, many people have been, they have been shaken out of their life. So, when they come out from jail, they now start taking drugs to be showing they are masculine, that they are no longer disturbed” (FGD1, participant #8). Another participant made the explicit link between different syndemic factors: “When you are discriminated, you get depressed. Then, when you are discriminated, you fall down to using drugs to make yourself feel OK” (FGD2, participant #8).

Not only did participants readily make connections between syndemic factors through direct pathways described above, but they also sometimes saw connections that reversed the order in which syndemic factors connected. For example, substance use heightening material insecurity, or material insecurity facilitating discrimination. One participant recounted how material insecurity necessitated he and his partner share a single room, which made it easier for the neighbors to hear them when they fought, and in turn to call the police on them (FGD1, unidentified participant). In particular, multiple participants indicated that money allowed freedom of expression and freedom from the scrutiny of police and society more generally. Participants from the TGW FGD were more likely to highlight the freedom associated with material security. Money in particular made it possible to pay bribes requested by police: “Most of the police that accept bribe, when you don’t have money, you will not be able to cover everything up” (FGD3, participant #1). Money also provided recourse in the event of familial repudiation: “When my family members find out who I am, what covers me up [protects me] was that I am not depending on them.. If tomorrow, I leave the family and go to another environment, I can feed and take care of myself. I can defend myself and I will focus my life on the way I want to live” (FGD3, unidentified participant). As another participant summarized the relationship, “For our country today, if you have money, you can live the life that you want” (FGD3, participant #8).

### Co-Prevalence and Sensitivity Analyses

Using the quantitative data to examine co-prevalence of pairwise syndemic components identified in our qualitative analyses, nearly every single pairwise co-prevalence was significantly greater than expected (Table 5). However, compared to the quantitative findings, qualitative discussions did not mention intimate partner violence as much as may have been expected. Additionally, our sensitivity analyses collapsing substance use into one factor, and discrimination into a single factor, did not change results in a substantial way (less than a 5% difference in regression estimates, with associations remaining statistically significant).

**Table 5:** Prevalence and pairwise co-prevalence of syndemic factors among sexual and gender minority people in Abuja, Nigeria (*n* = 515)

	Total	SDP	MatI	IPV	Stim	Dep
SGMD	20.2%	**11.7%**	**7.6%**	**11.1%**	**5.1%**	**8.2%**
SDP	16.7%	–	**6.4%**	**7.4%**	**3.9%**	**6.2%**
MatI	26.2%	–	–	**8.7%**	**5.8%**	**12.6%**
IPV	19.0%	–	–	–	**3.7%**	**9.1%**
Stim	12.4%	–	–	–	–	2.9%
Dep	26.0%					–

*SGMD* = Sexual/gender minority discrimination above or equal to third quartile, *SDP* = SGM discrimination from police above third quartile, *MatI* = Material insecurity above or equal to third quartile, *IPV* = Any intimate partner violence, *Stim* = Any stimulant use, *Dep* = Depression score above or equal to third quartile

Co-prevalence significantly (*p* <.05 using Chi-square test) greater than expected are bolded

## Discussion

We identified a syndemic characterized by discrimination, material insecurity, depression, substance use, intimate partner violence, and police and other violence among SGM in Abuja, Nigeria. Our quantitative analyses found strong relationships between this syndemic and positive HIV status, both before and after adjustment for sociodemographic characteristics. Our qualitative findings provided further evidence for relationships between components of the syndemic, with participants describing how these components interconnected in their lived experiences to impact their health and well-being. Overall, our study aligns with much of the literature demonstrating the relevance of a syndemic framework to understanding HIV-related health outcomes among SGM [6, 8, 16, 27, 28]. A unique exception was the limited discussions of intimate partner violence in the FGD. This absence does not necessarily indicate the absence of such violence, as it is likely due to the heavily vulnerable and stigmatized nature of this topic, making it extremely difficult to raise in the presence of others in a FGD. But, the quantitative analysis supports the relevance of intimate partner violence in the identified syndemic, which identified positive relationships between intimate partner violence and the other components of the syndemic.

Although discrimination, material insecurity, depression, substance use, intimate partner violence, and police and other violence can independently result in greater vulnerability to HIV, these factors co-occur in ways that amplify one another and subsequent behaviors that increase the risk of HIV acquisition [16, 27, 28]. In our focus groups, participants described how material insecurity can lead to depressive symptoms, which leads to various forms of substance use as a means of coping. Participants noted discrimination based on SGM identity as especially impactful, particularly when expressed by family members, church communities, and law enforcement. This discrimination led to feeling hopeless and depressed, with subsequent substance use. Many of these findings highlight the importance of structural factors on individual-level components of the syndemic. Community and societal-level systems, such as churches and police, have a direct impact on individual-level behaviors and experiences through systematic discrimination [11]. The range of discrimination experiences from many sources, which functioned differently in their day-to-day lives, supports keeping these multiple sources of discrimination as distinct components of the syndemic. These factors are especially relevant in the context of Nigeria, where discrimination against SGM people is culturally acceptable and explicitly coded into law [11, 29]. Thus, addressing this syndemic, and the vulnerability to HIV acquisition and other health issues it creates, requires systemic changes to both Nigerian cultural norms and laws, as well as other efforts to reduce discriminatory actions by religious leaders, healthcare workers, and the police.

Overall, our study highlights the importance of developing structural and individual interventions that target syndemic components that co-occur, such as material insecurity and depression, or depression and substance use. For example, an intervention addressing material insecurity and depression could include depression screening and support groups [30] in conjunction with job training. It is imperative to continue resourcing SGM-friendly health services for both sexual health and broader health as well as other mechanisms to improve their wellbeing. Changing Nigeria’s criminal code to decriminalize same-sex sex and/or repealing the 2013 Same-Sex Marriage (Prohibition) Act would beneficially alter the social and legal structure, removing symbolic and legal support for discrimination. Nigerian groups have attempted such legal efforts, albeit with minimal success [31]. Training to reduce discriminatory behavior among police officers would also be beneficial [32], as would increased pay for police officers to reduce extortion and bribery of SGM. Structural interventions have the ability to bring about more durable change, but individual interventions are also relevant. In particular, low-cost mental health interventions such as the group-based interpersonal therapy used by StrongMinds International among non-SGM populations elsewhere in Africa [33, 34] have the potential to reduce depression among SGM populations. In addition, interventions targeting substance use will also have reverberating effects on syndemic factors, reducing material insecurity and interactions with the police.

Our study has several strengths that bolster the significance of our findings. We utilized a mixed methods approach that triangulated strong quantitative relationships among our syndemic components and HIV status with qualitative themes that described these social and structural interrelationships. The use of a mixed methods approach provides additional qualitative nuance and description that our quantitative findings alone would not achieve. We also utilized a large range of factors, most of which include multidimensional items, capturing a broad set of relevant syndemic components. Finally, our study fills an important gap in the literature on how a multi-level syndemic is associated with HIV status and health seeking behavior among SGM in Abuja, Nigeria, one of the largest cities in Nigeria.

Our findings also have important limitations to consider. Our study population is SGM living in Abuja, Nigeria, so our findings may not be fully generalizable outside of this context. This focus is justified however, given both the disproportionate vulnerability to HIV among SGM, and the greater relevance of some of our syndemic components in Nigeria, particularly discrimination towards SGM, which is both legally and culturally enforced in Nigeria. The relatively small number of TGW in our sample limits our understanding of the identified syndemic in this population, indicating an important area for future research. Some factors, such as stigma and discrimination, had much more granular information than our single-item measures, such as intimate partner violence. There are additional factors of interest related to HIV acquisition in this population, such as transactional sex, that we were unable to include in our analyses but are important for future research. Finally, given the sensitive nature of the topics discussed, social desirability is likely to have resulted in some underreporting, particularly in the FGD, where experiences are shared among peers. Despite the risk of social desirability, participants shared many vulnerable experiences, in part due to a strong rapport with one another, the data collectors, and the TRUST clinic.

## Conclusion

We found strong evidence of a syndemic of discrimination, material insecurity, depression, substance use, intimate partner violence, and police and other violence among SGM in Abuja, Nigeria. Using a mixed-methods approach, we found statistically significant associations between our syndemic index and positive HIV status, and provided nuanced descriptions of how the component factors of the syndemic interrelate to lead to adverse health outcomes among this population. Discrimination, material insecurity, and depression are especially salient to the lived experiences of SGM in Abuja, Nigeria. This multilevel syndemic has a significant impact on the physical, mental, and emotional health of this community. We recommend future research assessing this syndemic overall and temporal relationships with additional health outcomes in this population. Similarly, future research exploring this syndemic among TGW in Nigeria will further help inform health equity efforts for this community. Overall, our findings highlight important needs and disparities that must be addressed not simply at the individual level, but through larger scale social and structural change efforts.
